# Macrophage Motility in Wound Healing Is Regulated by HIF-1α via S1P Signaling

**DOI:** 10.3390/ijms22168992

**Published:** 2021-08-20

**Authors:** Islamy Rahma Hutami, Takashi Izawa, Tsendsuren Khurel-Ochir, Takuma Sakamaki, Akihiko Iwasa, Eiji Tanaka

**Affiliations:** 1 Department of Orthodontics and Dentofacial Orthopedics, Institute of Biomedical Sciences, Tokushima University Graduate School, Tokushima 770-8504, Japan; rahma.hutami@unissula.ac.id (I.R.H.); ktsendsuren@gmail.com (T.K.-O.); c301851004@tokushima-u.ac.jp (T.S.); iwasa.akihiko.1@tokushima-u.ac.jp (A.I.); etanaka@tokushima-u.ac.jp (E.T.); 2 Department of Orthodontics, Faculty of Dentistry, Sultan Agung Islamic University, Semarang 50112, Indonesia; 3 Department of Orthodontics, Graduate School of Medicine, Dentistry and Pharmaceutical Sciences, Okayama University, Okayama 700-8525, Japan

**Keywords:** HIF1, M1/M2 macrophage, S1P, wound healing

## Abstract

Accumulating evidence indicates that the molecular pathways mediating wound healing induce cell migration and localization of cytokines to sites of injury. Macrophages are immune cells that sense and actively respond to disturbances in tissue homeostasis by initiating, and subsequently resolving, inflammation. Hypoxic conditions generated at a wound site also strongly recruit macrophages and affect their function. Hypoxia inducible factor (HIF)-1α is a transcription factor that contributes to both glycolysis and the induction of inflammatory genes, while also being critical for macrophage activation. For the latter, HIF-1α regulates sphingosine 1-phosphate (S1P) to affect the migration, activation, differentiation, and polarization of macrophages. Recently, S1P and HIF-1α have received much attention, and various studies have been performed to investigate their roles in initiating and resolving inflammation via macrophages. It is hypothesized that the HIF-1α/S1P/S1P receptor axis is an important determinant of macrophage function under inflammatory conditions and during disease pathogenesis. Therefore, in this review, biological regulation of monocytes/macrophages in response to circulating HIF-1α is summarized, including signaling by S1P/S1P receptors, which have essential roles in wound healing.

## 1. Introduction

Hypoxia-inducible factor (HIF) is a transcription factor that is composed of two basic helix-loop-helix proteins, α and β, which both belong to the PER-ARNT-SIM (PAS) family [1]. Under hypoxic conditions, HIF binds to a pentanucleotide sequence (RCGTG) present in hypoxic response elements (HREs) that are carried by certain target genes. All three subunits of HIF-α—1α, 2α, and 3α—are highly responsive to hypoxic conditions. There are only two types of β subunits, aryl hydrocarbon receptor nuclear translocator (ARNT, also known as HIF-1β) and ARNT2. The latter is expressed in the nucleus, is not responsive to oxygen, and has other functions in gene transcription [2,3,4,5,6].

The roles and functions of HIF-1α and HIF-2α have been more extensively studied than those of HIF-3α. Five variants of HIF-1α have been reported to be associated with its exon structure or amino acid sequence: HIF-1α^557^, HIF-1α^736^, HIF-1α^FL^ (or HIF-1αZ), HIF-1α^516^, and HIF-1α^785^ [7,8,9,10]. For several different functions, HIF-1α and HIF-2α have overlapping roles. However, HIF-2α is characterized by more restricted expression, is also known as endothelial PAS domain protein 1 (EPAS1), and has a smaller structure than HIF-1α [11,12]. HIF-3α has a smaller transactivation domain than both HIF-1α and HIF-2α. In humans, six HIF-3α variants have been reported, HIF-3α1–6 [12,13]. 

In macrophages, HIF-α isoforms can be differentially activated, and they contribute to regulation of macrophage polarization depending on their microenvironment. Macrophages often localize to hypoxic tissues, and this environment can strongly affect essential macrophage functions [14,15]. During M1 and M2 macrophage polarization that is induced by Th1 and Th2 cytokines, respectively, mRNA levels of *HIF-1α* and *HIF-2α* are differentially expressed. HIF-1α and HIF-2α further contribute to M1 and M2 macrophage functions by binding inducible nitric oxidase synthase (iNOS) or arginase-1, respectively, to maintain nitric oxide (NO) homeostasis during inflammation [15,16,17]. 

Sphingosine-1-phosphate (S1P) plays a role in the activation of macrophages and is subsequently produced in and affects macrophage functions. The role of S1P under hypoxic conditions has been extensively investigated [18,19,20]. S1P production, S1P receptor (S1PR) expression, and signaling mechanisms mediated by both have been shown to be dynamic. During inflammation, higher levels of S1P are present in affected tissues, thereby leading to greater signal input to macrophages from their environment [21]. S1P also acts to guide macrophages in their migration to a site of inflammation and injury. Moreover, S1P-dependent migration has been found to be strictly dependent on the S1PR profiles [22]. 

Recent evidence showed that targeting the S1P/S1PR signaling pathway is crucial for the treatment of immune-mediated diseases (multiple sclerosis and rheumatoid arthritis) [23,24,25], inflammatory bowel diseases [26], lung diseases [27,28], liver diseases [29,30], vascular diseases [31,32,33], brain diseases [34,35], renal diseases [36], and allergy [37]. In the latest clinical trial research, S1P/S1PR also act as a potential new adjuvant therapy to alleviate viral infection of COVID-19 symptoms [38]. In addition, the long duration treatment has been observed to be effective by targeting S1P/S1PR signals in fingolimod (FTY720) [34], KRP-203 [39], and ozanimod (RPC1063) [26]. However, to establish the long-term effect by targeting S1P/S1PR in chronic diseases, larger samples and longer treatment duration for assessing clinical efficacy and safety remain to be studied.

The importance of the S1P/S1PR signaling axis is demonstrated in knockout (KO) mouse models. Amongst all S1PRs, S1P_1_ is the most critical S1P receptor for angiogenesis, while S1P_2,3_ is redundant or enhancing S1PR signaling function [40]. The defect also occurs in the nervous system development or hinders lymphocytes migration [40,41]. Identification of S1P_4,5_ in the KO mouse model suggested that S1P_4_ receptors play a role in the essential regulation of megakaryocytes morphology and platelet repopulation, while S1P_5_ acts as an oligodendroglial receptor that regulates oligodendrocytes development [42,43]. Disturbance during vascular development caused by a defect in the S1P/S1PR signal affects vascular integrity and coagulation processes [44]. An imbalance in the S1P gradient causes S1P to play the role of both a pro- and an anti-inflammatory agent in various immune cells, such as monocytes/macrophages, platelets, mast cells, lymphocytes, endothelial cells, and fibroblasts [45].

**Figure 1:**
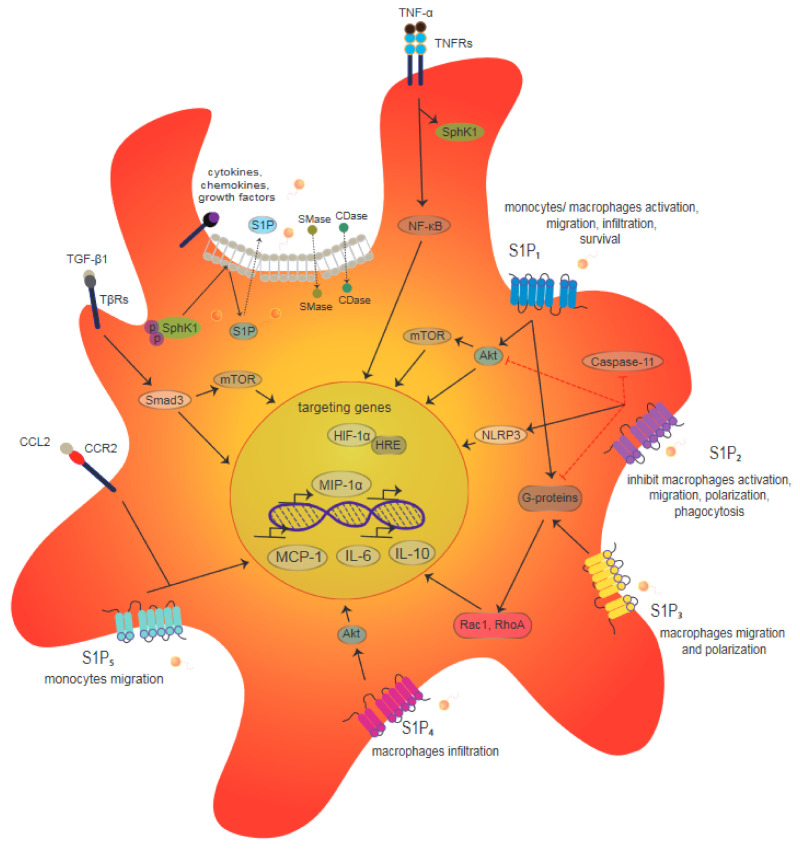
Role of S1P/S1PRs in macrophages. S1PRs on macrophages can be activated by extracellular molecules such as cytokines, chemokines, or growth factors. Receptor activation transmits a signal for sphingomyelinase (SMase) and ceramidase (CDase) to sequentially breakdown sphingomyelin and ceramide, respectively, to generate sphingosine. Phosphorylation (p) of the latter by Sphk1 produces S1P, which induces intracellular signaling to activate kinase-mediated phosphorylation of SphK1. Translocation of S1P from the cytosol to the cell membrane also leads to phosphorylation of sphingosine to S1P. SphK1 is essential for the TNF signaling pathway for downstream molecule NF-κB activation. Akt signaling molecules are activated by S1P_1_ and S1P_4_ and signal for macrophage activity and survival such as MCP-1 and MIP-1α. G protein through Rac1 and RhoA activation plays a role in S1P_1_ and S1P_3_ as the regulators of macrophage migration and infiltration. To the contrary, the migration signals are inhibited by S1P_2_, by which NLRP3 becomes a considerable signal to shade the S1P_2_ blockade mechanism to inhibit the macrophage death signal, caspase-11. Meanwhile, S1P_5_ signal activation regulates monocytes migration activity together with CCR2. TGF- β signaling pathway through molecules downstream of Smad3, Akt, and mTOR Smad3 mediates HIF-1α stabilization, and expression thus activates targeting genes containing HRE.

Macrophages all express S1PRs (S1P_1–5_), although their expression profile varies according to subtype and their distinct functional properties (Figure 1) [46]. For example, in peritoneal macrophages, S1P_1_ acts as a pro-migratory signal involving Rho kinase and PI3K-Akt1 [21,47]. Conversely, S1P_2_ signaling stimulates cAMP production to inhibit macrophage migration via NLRP3 inflammasome activation, thereby attenuating phosphorylation of Akt in peritoneal inflammation [30,48]. Meanwhile, S1P_3_ has been shown to promote activation of inflammatory macrophages in microglia, while a model of brain ischemia has demonstrated that S1P_3_ regulates expression levels of inflammatory genes (e.g., *IL-6*, *IL-1β*, *TNF-α*, *iNOS*, and *COX-2*) via induction of lipopolysaccharide (LPS) [49,50]. To date, S1P_4_ and S1P_5_ remain less studied compared with S1P_1–3_. S1P_4_ is expressed by leukocytes, T cells, monocytes, and macrophages [51,52]. Under pro-inflammatory conditions, macrophages downregulate S1P_4_, while S1P_4_ levels are higher on M2-polarized macrophages [53]. Furthermore, on T cells, S1P_4_ contributes to suppression of cellular proliferation by reducing expression of interleukin (IL)-2 and interferon (IFN)-γ and increasing levels of IL-10, while not affecting T cell migration [51,54]. Regarding S1P_5_, it contributes to the exit of monocytes from bone marrow and tissue macrophages during inflammation [55]. 

In order to survive hypoxic conditions during inflammation, macrophages need to migrate to specific tissue sites. Recent studies of HIF-1α have highlighted the important role of S1P/S1PR signaling for this migration phenotype [52,53,56,57,58]. However, the mechanistic details regarding the communication that takes place between these signaling pathways in macrophages remain to be elucidated. Therefore, in this review, HIF-1α and S1P/S1PR signaling in macrophages in response to inflammation in wound healing is presented, and insights into possible mechanisms are discussed.

## 2. Biological Regulation of HIF-1α in Macrophage

### 2.1. The Cellular Regulation of HIF-1α

Under normal oxygen conditions, HIF-1α proteins are continuously synthesized and degraded via the proteasome pathway [59,60,61]. As a result, HIF-1α has a very short half-life. However, in the presence of decreasing concentrations of oxygen, degradation of HIF-1α is reduced. This oxygen-dependent regulation is achieved due to an oxygen-dependent degradation domain (ODDD) in HIF-1α that contains Fe^2+^ and two key prolyl residues (Pro^402^ and Pro^564^). When these prolyl residues undergo oxygen-dependent hydroxylation, HIF-1α is recognized by a component of an E3 ubiquitin ligase complex, von Hippel–Lindau tumor suppressor protein (pVHL), and targeted for degradation via the ubiquitin-proteosome pathway [62,63,64,65,66,67,68,69,70]. Therefore, when oxygen levels are low in a hypoxic environment, HIF-1α exhibits a longer half-life.

While pVHL has been shown to have a vital role in controlling HIF-1α stability via ubiquitination [71], a pVHL-independent pathway also exists. The latter is mediated by tumor suppression protein p53 and forkhead box O4 (FOXO4) and promotes HIF-1α proteosomal-mediated proteolysis [72,73]. Other components of this proteolysis complex include elongin-B, elongin-C, Cul2, and Rbx1, which are components of other E3 ubiquitin ligase complexes as well. Degradation of HIF-1α effectively blocks transcriptional activation of its downstream genes under normoxic conditions. In contrast, under hypoxic conditions, prolyl hydroxylation is suppressed and HIF-1α accumulates and translocates to the nucleus. After HIF-1α forms a dimer with HIF-1β, this trans-activating complex is able to bind HREs or enhancer sequences present in target genes. Notable target genes include vascular endothelial growth factor (VEGF), erythropoietin, iNOS, heme oxygenase-1, enolase-1, aldolase-A, lactate dehydrogenase-A, and phosphoglycerate kinase-1 [2,74,75]. HIF-1α signaling pathways have also been shown to influence collagen-prolyl-4-hydroxylase, urokinase-type plasminogen activator receptor, matrix metalloproteinase-2 (MMP2), and tissue inhibitor of MMP (TIMP)-1 [76]. 

VEGF is directly regulated by HIF-1α [77], and the biological activity of VEGF is further enhanced when VEGF receptor-1 (VEGFR-1/Flt-1) is upregulated in response to hypoxia. *VEGF* mRNA also exhibits greater stability under hypoxic conditions [78]. In endothelial cells, there is an autocrine signaling loop that involves signaling through VEGF and VEGFR-1 to provide hypoxic induction of VEGFR-2. When HIF-1α is deleted, impaired vascularization of xenografts has been observed [79]. The phosphatidylinositol 3-kinase (PI3K)/Akt pathway has also been shown to affect HIF-1α/VEGF expression in the presence of vanadium, a carcinogenic agent, via reactive oxygen species (ROS) [80]. 

It is known that hypoxic conditions affect oxidative stress. There are several sources of ROS, including the respiratory electron transport chain in mitochondria, the family of NADPH-oxidase enzymes, xanthine oxidase, peroxisomes, cytochrome p450 enzymes, cyclooxygenase, and lipoxygenase [81]. Mitochondria are influenced by HIF-1α during the cellular respiration process. Under hypoxic conditions and in response to HIF-1α, the composition of the cytochrome oxidase complex is changed [76,82]. Moreover, mitochondria-derived ROS that are produced by electron transport chain complex III stabilize HIF-1α [82]. ROS also increase the transcriptional activity of HIF-1α [83]. Experimentally, this was demonstrated when cells incubated with H_2_O_2_ exhibited a longer half-life for HIF-1α and genes targeted under normoxic conditions were activated [84]. With greater stability, HIF-1 induces the mitochondrial protease LON, which is required for degradation of cytochrome c oxidase (COX)4-1 [76] and expression of pyruvate dehydrogenase kinase (PDK). The latter inhibits pyruvate dehydrogenase via phosphorylation. If pyruvate is not converted into acetyl CoA, pyruvate cannot enter the TCA cycle, and consumption of mitochondrial oxygen is reduced. ROS generation in response to hypoxia is also attenuated [76,85,86].

In keratinocytes, when *SphK1* is deleted, ROS production is drastically reduced via NF-κB, as well as its anti-inflammatory effects via phorbol 12-myristate 13-acetate [87]. Deleted SphK1 also inhibits the accumulation of ceramides via S1P, which leads to ROS enhancement in non-alcohol liver injury [88]. N-acteylcysteine, a ROS scavenger, suppresses HIF-1α and VEGF via blockade of SphK1 activity under hypoxic conditions [89], while SphK1-dependent stabilization of HIF-1α is mediated via the Akt/GSK3β signaling pathway [90]. Cho et al. revealed a role for SphK1 in melantonin-induced inactivation of HIF-1α under hypoxic conditions [91]. Melantonin suppresses Akt/GSK3β and ROS-related signaling to decrease SphK1 activity and thereby decrease stabilization of HIF-1α expression [91]. Neutralization of ROS by antioxidants is known to abolish induction of HIF-1α during hypoxia [92,93]. HIF-α directly modulates ROS expression by inhibiting prolyl hydroxylases or their cofactors [94], and it indirectly modulates ROS expression by activating signaling upstream of HIF-α (e.g., mitogen-activated protein kinases (MAPK) [95,96], PI3K/Akt, and ERK-induced Rac1 [80,95,96,97,98]). 

There are several molecules downstream of PI3K/Akt that regulate levels of HIF-1α protein. These include mammalian target of rapamycin (mTOR), glycogen synthase kinase-3-β (GSK3β), FOXO3, and Bcl-2 apoptosis-related family members [99,100,101,102]. GSK3β has been shown to contribute to the destabilization of HIF-1α in a VHL-independent manner [98,103,104]. Meanwhile, Akt inactivates phosphorylation of (p)-GSK3β in its ODDD and promotes HIF-1α accumulation [103]. Therefore, it is hypothesized that HIF-α and ROS activity are controlled via protein kinase phosphorylation, potentially through the universal phosphorylation signal transduction pathway of PI3K/Akt [105].

During cell cycle regulation, mTOR is a hypoxic sensor and a target of Akt. As an upstream mediator of HIF-1α activation, mTOR can also alter HIF-1α post-transcriptionally [106]. Previous studies have further revealed that signaling through the PI3K/Akt pathway via mTOR/FKBP-rapamycin-associated protein (FRAP) signaling pathways is regulated by HIF-1α [107]. Correspondingly, in the presence of individual inhibitors of PI3K and FRAP, or a dual inhibitor of PI3K/mTOR (LY294002, rapamycin, and NVP-BEZ235, respectively), activation of p-Akt is suppressed, and this is followed by reductions in expression levels of HIF-1α and VEGF under hypoxic conditions [106,107,108]. Wortmannin, a specific inhibitor of the PI3K/Akt pathway, has also been shown to inhibit expression of HIF-1α [109]. Thus, targeting of PI3K/Akt signaling pathways has led to repression and then sensitization to cellular death via HIF-1α gene expression [110]. 

### 2.2. Role of HIF-1α in Inflammation and Immune System Regulation Involving Macrophages

Macrophages represent one of the first barriers of the immune system that invading pathogens encounter [111,112,113]. Macrophages are able to engulf pathogens, including phagocytic dead cells and cellular debris, and perform degradation in phagolysosomes [114,115,116,117]. Macrophages also employ an array of direct antimicrobial mechanisms by generating ROS and reactive nitrogen species in phagosomes, delivering cathepsin and maturing phagosomes via hydrolase [118,119,120,121]. Therefore, the importance of ROS in macrophages acting as immune cells is well recognized. 

Regarding the regulation of ROS in mitochondria, increased ROS expression catalyzes the PHD2 domain, thereby impairing its activity and inducing specific post-translational modifications. This inhibition of PHD2 subsequently promotes HIF activation to affect pro- and anti-inflammatory activities to regulate immune cells [16,122,123]. Macrophages, upon stimulation, can change their metabolic activity to acquire a phenotype that is usually associated with a pathological immunological niche. Consequently, decreases in local O_2_ can create a hypoxic microenvironment that leads to activation of HIF signaling and modulation of immune cell activity [124]. Thus, a correlation exists between hypoxic conditions, inflammation, and immune cell metabolism. 

Hypoxic conditions stimulate the activity and survival of macrophages. Briefly, HIF activation induces ATP production, which increases cellular motility, invasiveness, and bactericidal activity [125,126]. Treatment of macrophage with LPS has been shown to upregulate pro-inflammatory genes, such as *IL-10*, *IL-1β*, *HIF-1α*, and *NF-κB p65* [127]. Meanwhile, Aarup et al. have provided in vivo evidence that macrophages lacking HIF-1α in atherosclerosis mice exhibit decreased apoptosis, migration, and glucose uptake [128,129]. Conversely, overexpression of HIF-1α induces the polarization of pro-inflammatory macrophage (M1) through NF-κB to upregulate genes related to glycolysis metabolism (e.g., *PDK1*, *PGK1*, *GLUT1*, *GCK*, and *PKM2*) [130]. 

In vitro, M1 macrophage have been stimulated with intracellular proteins and nucleic acids from lysed cells, as well as with bacterial components (IFN-γ, LPS, and peptidoglycan, respectively) [131]. M1 macrophages are characterized by production of NO, iNOS, ROS, IFN-γ, IL-6, IL-1β, IL-12, CD86, CD80, MHC-II, and tumor necrosis factor (TNF)-α. They also secrete MMP-2 and MMP-9 for degradation of the extracellular matrix to facilitate cell migration and infiltration, and they produce growth factors such as platelet-derived growth factor, insulin-like growth factor-1, VEGF, and tumor growth factor (TGF)-β1 for cell proliferation, granulation, tissue formation, and angiogenesis [132,133,134,135,136].

HIF-1α expression is independent of NF-κB. On the other hand, the secondary target genes of NF-κB activation facilitate the chromatin remodeling of nucleosome of IkBζ (*Nfkbiz*) [137], and they may associate with the M1 phenotype. However, both the HIF-1α and HIF-2α subunits appear to be essential for maintaining levels of NF-κB p65 [100,138]. It appears that HIF-2α may promote anti-inflammatory and pro-resolving/regenerative M2 macrophages [17,139]. However, incongruent roles for HIF-2α have been reported. For example, HIF-2α promotes IL-1β expression [140] and competes with iNOS for arginine metabolism, thereby limiting synthesis of NO [17]. The latter is associated with an M1 phenotype rather than an M2 phenotype [140]. Thus, HIF-1α and HIF-2α appear to have overlapping functions, yet they also have distinct functions that cannot be compensated for by the other [139]. This spectrum of phenotype activation, which at times can be redundant, requires further study.

**Figure 2:**
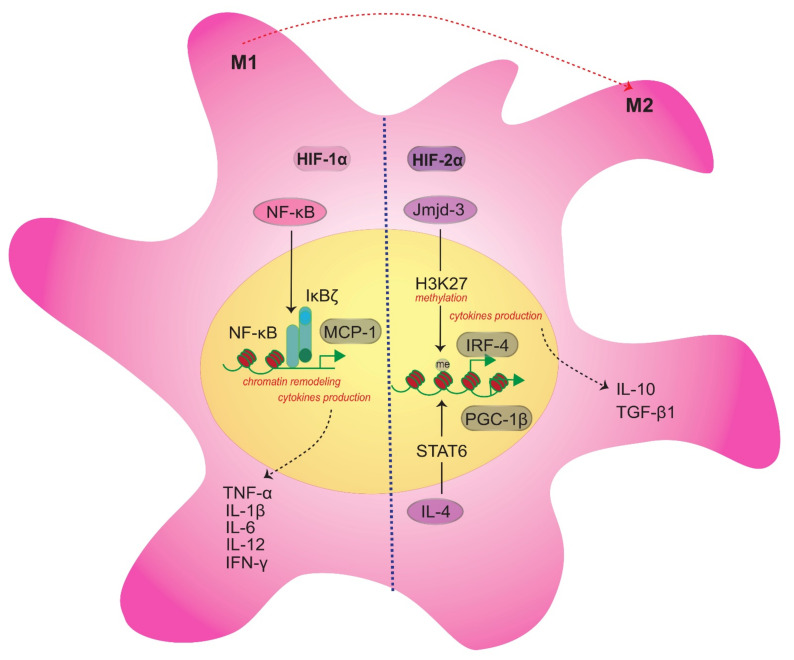
Macrophage intracellular reprogramming during polarization. Balanced switching of M1/M2 macrophage action is crucial. Both the HIF-1α and HIF-2α subunits are essential to maintain the NF-κB level. However, due to the incongruent roles of HIF-2α, the part of the phenotypic switch between HIF-1α and HIF-2α in M1/M2 macrophages remains uncertain. M1 macrophage polarization is NF-κB-independent. NF-κB mediates the IkBζ recruitment to target promoter and acts as a transcriptional coactivator for chromatin remodeling that regulates the CCL2 (MCP-1) gene, thus producing cytokines such as TNF-α, IL-1β, IL-6, IL-12, and IFN-γ. M2 macrophages upon IL-4 stimulation activate STAT6, which then induces PGC-1β that is essential for the M2 macrophage profile and is characterized by IL-10 and TGF-β1 expression. Meanwhile, Jmjd-3, a histone 3 Lys27 (H3K27) demethylase that directly targets IRF-4, is crucial for M2 macrophages.

M2 macrophages reduce inflammation via upregulation of IL-10 expression, arginase-1, programmed death-ligand-2, resistin-like molecule-α, and TGF-β1. However, M2 macrophages also remodel and strengthen the extracellular matrix in the presence of MMP-12 and MMP-13 expression [134,141]. Additionally, M2 macrophages can be stimulated by IL-4 and IL-13, and they express mannose receptor CD206, dectin, IL-10, and TGF-β1 [131]. Upon IL-4 treatment, transcription factor STAT6 is activated, which then induces peroxisome proliferator-activated receptor (PPAR)γ-coactivator (PGC)-1β. PGC-1β subsequently induces biogenesis and mitochondrial respiration and further interacts with estrogen-related receptor-α and nuclear respiratory factor-1 to drive synthesis of cytochrome c and ATP [142,143]. When PGC-1β is knocked down, both the function and metabolic profile of M2 macrophages are impaired [144]. Jumonji domain-containing (Jmjd)-3 is a histone 3 Lys27 (H3K27) demethylase that has a role in macrophage activation that is NF-κB-dependent following stimulation of TLR [145]. The transcription factor IRF4 is crucial for the M2 macrophages response and is a direct target of Jmjd3-mediated demethylation [146]. Therefore, transcription factors may represent key metabolic switches in M2 macrophages (Figure 2).

## 3. The Role of S1P/S1PR in Macrophages during Inflammation

S1P and ceramide are potential bioactive lipid mediators that regulate cellular pleiotropic activities such as survival, proliferation, inflammation, and migration [147,148,149]. S1P is a pro-apoptotic backbone component of all sphingolipids [150] and is generated from ceramidase and sphingosine kinase (SphK). Correspondingly, SphK is a central component of sphingolipid metabolism, which both synthesizes and degrades sphingolipids [151]. Briefly, de novo ceramide synthesis initially combines palmitoyl-CoA and serine to form 3-keto-dihydrosphingosine, which is subsequently reduced to dihydroceramide [152]. A desaturate subsequently generates the corresponding ceramides that can undergo phosphorylation or glycosylation to form glucosylceramides. The latter are further processed to form glycosphingolipids that are presented at the plasma membrane. Alternatively, ceramides can be converted to sphingomyelin and incorporated into the outer cell membrane. If attacked by acidic or neutral sphingomyelinases, these sphingomyelins are converted back to ceramide. Following cleavage by ceramidases, the ceramides form sphingosine, which can be phosphorylated by two sphingosine kinase isoforms, SphK1 and SphK2, to generate S1P [45,153,154]. 

S1P is a ligand of five high-affinity (S1P_1–5_) G protein-coupled receptors that are linked to either G_i_, G_q_, and/or G_12/13_. These receptors exhibit distinct tissue expression profiles, and their specific effects are dependent on these profiles [155,156,157]. Myeloid cells express G_12/13_, G_q/13_, and G_s_ [158]. G_12/13_ belongs to the α-subunit that shares 67% amino acid sequence identified, and it stimulates an effector pathway of GTPase RhoA [159,160]. S1P is produced intracellularly by Sphk1 that is activated in response to several stimuli, including pro-inflammatory cytokines [45]. It is hypothesized that S1P induces activation of inhibitor of κ kinase (IKK)-β and c-jun amino-terminal kinase (JNK) via upstream activation of TGF-β-activated kinase-1. SphK-1 can also block JNK activation and prevent inflammation, while inhibition of SphK1 leads to activation of JNK. Interestingly, S1P opposes the effects of ceramide by counteracting ceramide-induced activation of JNK [149,161]. Thus, the ratio of ceramide-to-S1P may function as an intracellular rheostat.

S1P can exert both paracrine and autocrine effects following secretion by the ATP-binding cassette transporter or the S1P transporter spinster homolog-2 [162,163,164]. In circulation, S1P is characterized by a high nanomolar concentration [165]. Recent studies have reported that S1P mediates intracellular functions, specifically histone acetylation in the nucleus [166]. If confirmed, a link between S1P and epigenetic regulation of gene expression would be established [166]. 

S1P has been shown to act as a cellular motility regulator in conjunction with TGF-β/Smad3 signaling to maintain cartilage homeostasis in osteoarthritis [167]; S1P also acts as a cofactor for TNF receptor-associated factor-2 to produce ubiquitin ligase activity for activation of NF-κB [168]. Activated NF-κB is then able to interact with prohibitin-2 and mediate mitochondrial respiration [169]. It may also modulate p21-activated kinase-1 activity [170]. Ishii et al. revealed that S1P/S1P_1_ interactions regulate the egress of osteoclast precursor cells from circulation to bone tissues [171]. Meanwhile, S1P_2_ modulates NFATc1 to affect osteoclastogenesis and pro-inflammatory cytokines [172]. The S1P_2_-mediated caspase-11 p26 subunit is also known to induce macrophage death under sepsis conditions [173]. Dying cells release S1P, which activates S1P_1/3_ in macrophages to upregulate COX-2 triggering of VEGF [174]. Moreover, Fas apoptotic signaling promotes osteoclast precursor cells or bone marrow macrophages (BMMs)-induced osteoclasts, in conjunction with S1P_1_ signaling through NF-κB p50 subunit activation in rheumatoid arthritis [23,175]. Thus, targeting components downstream of S1PR signaling may represent a promising therapeutic approach.

Signaling through S1P/S1PRs promotes M1/M2 polarization of macrophages by affecting cytokine production and migration phenotype [21,53]. Different subtypes of macrophages exhibit distinct SIPR profiles, yet all macrophages express all five S1PRs to some extent [53]. Moreover, receptor expression profiles appear to correspond to distinct functional properties (Figure 1) [46]. Under inflammatory conditions, levels of S1P increase, and this increase is sensed by various types of cells, including macrophages. Macrophages are exposed to multiple signals from their environment, and they adjust their response accordingly. It is hypothesized that inflammatory, homeostatic, or regenerative conditions, as well as S1P production and S1PR expression and/or signaling, further add to the complexity of the functional properties that characterize macrophage populations [21].

Inflammation is triggered by endogenous signals and microbial components such as LPS at sites of injury. Once established, sites of injury are characterized by paracrine recruitment, proliferation, and differentiation of circulating progenitor cells and diverse types of inflammatory cells [21]. Das et al. have demonstrated that activation of S1P/S1PRs during bone healing activates anti-inflammatory (M2) macrophages, promotes vascularization, and recruits bone marrow-derived mononuclear cells at the site of injury [176]. However, the specific types of S1PRs that regulate M1/M2 macrophages for various inflammatory or disease conditions remain unclear. It has been demonstrated that bacterial stimulation of macrophages leads to an increase in protein levels of S1P_3_ [177]. In the absence of S1P_3_, phagosome maturation of macrophages is affected via ROS activation [177]. When bone fractures have been treated with FTY720, a non-selective S1PR agonist, no improvement in healing was observed, and there was no difference in osteoclast numbers at the wound sites [178]. Yang et al. demonstrated that S1P_2_ and S1P_3_ have important roles in the polarization of M1 macrophages [179]. In both in vivo and in vitro studies, S1P-induced BMMs were observed to promote M1 macrophages, and this tightly correlated with expression of TNF-α and MCP-1 and the signaling pathway involving Gα_i/o_, PI3K, and JNK [179]. Various roles and actions of S1P/S1PRs signaling pathways in monocytes/macrophages are summarized in Table 1. Some of these studies explain the infiltration of M1/M2 macrophages under inflammatory conditions. For example, S1P_3_ knockout (KO) studies have shown that the number of M2 macrophages increases and fewer T cells infiltrate muscle wound sites [180]. In addition, it was observed that treatment with a S1P_3_ antagonist, VPC01091, improved tissue regeneration [180]. Decreased monocyte/macrophage motility detected in S1P_4_ [54] and SphK1 KO mice has also been associated with reduced S1P generation [181]. Under hypoxic conditions, SphK1-mediated accumulation of HIF-1α levels occurs and is dependent on Akt/GSK3β signaling [90]. In contrast, partial HIF-1α KO mice have been reported to aggravate the infiltration of M1/M2 macrophages via the S1P/S1P_1_ signaling axis [57]. Thus, overlap between the roles of S1P/S1PRs and HIF-1α signaling pathways may exist under inflammatory conditions and may also affect the function of M1/M2 macrophages.

## 4. Cellular Regulation of HIF-1α through S1P/S1P_1_ Signaling in Macrophages during Wound Healing

### 4.1. The Roles of Macrophage Profiles and Hypoxia on Wound Healing

In this section, the roles of HIF-1α and S1P in macrophages recruited to inflamed regions of wound healing are discussed. Specifically, activation, polarization, migration, and phagocytosis ability of these macrophages are discussed, as well as the pathways that potentially mediate these functions. Macrophages are present in all tissues. They are present as resident cells (Langerhans cells) or they are introduced as infiltrating monocyte-derived cells [182]. The tissue site is a predominant determinant of the phenotype of tissue-resident cells. The latter help to both maintain tissue homeostasis and act as sentinels of injury. Therefore, both recruited macrophages and tissue-resident macrophages substantially contribute to wound healing at a site of injury [183]. 

Wound healing is a tightly coordinated and highly dynamic process that is able to restore tissue integrity after hypoxia is induced with infection or physical trauma. As healing progresses, a decline in tissue hypoxia occurs. It has been demonstrated that hypoxia induces essential factors that stimulate the proliferation and migration of endothelial cells, macrophages, keratinocytes, and fibroblasts in wound areas [184,185,186]. There are three distinctive phases to the healing process: (1) coagulation and inflammation, (2) tissue formation, and (3) tissue remodeling [187]. In the first phase, a blood clot is established to provisionally close the wound. Concomitantly, recruitment of inflammatory cells is initiated [188]. In the subsequent tissue formation phase, cell proliferation is initiated by local growth factors, and pro-inflammatory signaling declines. Finally, the wound site is organized to restructure the tissue and complete the tissue remodeling phase [189]. In all three phases, macrophages are critical components [190]. 

It is possible that an imbalance in phenotype switching of M1/M2 macrophages induces tissue breakdown [191]. Furthermore, any cell depletion from monocyte or macrophage lineages will impair wound closure and the granulation of formation tissue [192]. Thus, macrophages fulfill distinct functional roles, and this highlights their diversity and plasticity in achieving these functions. 

### 4.2. Differential Wound Healing Is Accelerated at Skin and Mucosal Sites of Injury

**Figure 3:**
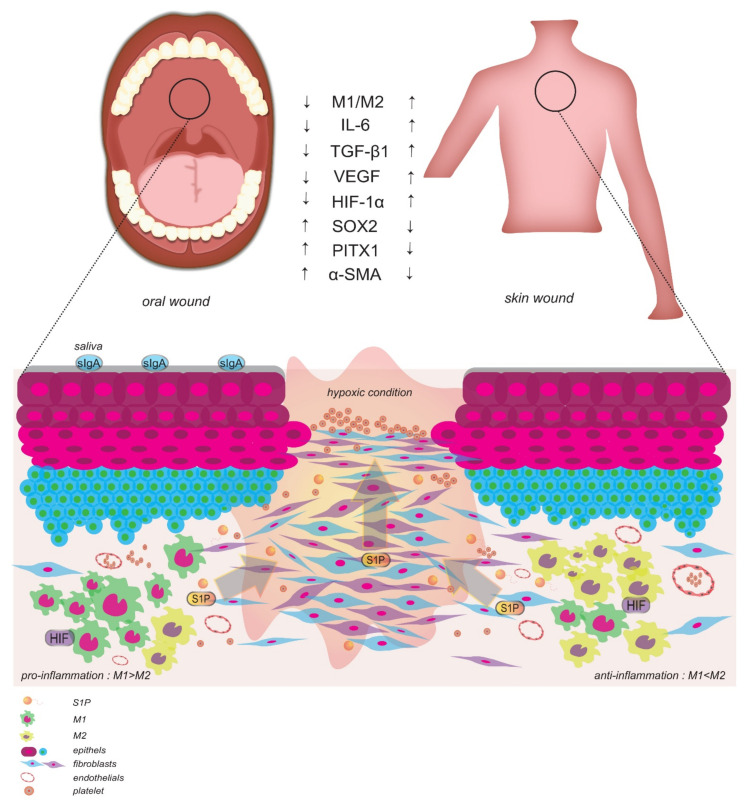
Role of metabolic reprogramming in oral wound healing under hypoxic conditions. Oral mucosal wounds heal faster than skin wounds, although having similar wound healing stages. The saliva is considered one factor that facilitates rapid mucosal wound closure by expressing secretory IgA. However, the essential cytokines, chemokines, growth factors, and protease inhibitors are interrelated and inseparable. Fewer M1/M2 macrophages and lower cytokine production (IL-6 and TGF-β1) during oral mucosal wound healing were observed. Lower VEGF expression found in the oral wound may be due to a decreased level of HIF-1α gene expression level, while higher expression levels of SOX2 and PITX1 followed by the increased level of α-SMA expression lead to acceleration and less scar tissue formation in oral wounds than in skin wounds. Wound S1P/HIF gradient cause S1P/HIF to act as pro- or anti-inflammatory agents for inflammatory cells such as M1/M2 macrophages.

It is well-documented that oral mucosal wounds heal faster than skin wounds, despite having the same stages of wound healing at each site (Figure 3) [186,193]. Multiple pro-inflammatory cytokines, chemokines, growth factors, and ROS production contribute to successful wound healing [194]. It has been observed in oral mucosal wounds that fewer inflammatory cells infiltrate the wounds (e.g., neutrophils, macrophages, mannose receptor-positive M2 macrophages, and T cells) [195], and cytokine production (IL-6 and TGF-β1) is reduced [193]. However, a robust increase in re-epithelialization is detected 24 h post-injury compared with skin wound closure [193]. It has also been observed that oral keratinocytes express lower levels of VEGF than skin keratinocytes under hypoxic conditions [196], and skin wounds exhibit higher levels of hypoxia and elevated levels of HIF-1α compared with mucosal wounds under stressed conditions [186]. Higher levels of HIF-1α expression in skin may be due to a greater abundance of gene expression related to the HIF-1α pathway in skin than in oral mucosa. Relevant genes include *MMP1*, *MMP8*, *MMP9*, *MMP10*, *MMP13*, *MMP23B*, *NOS3*, *SLC2A1*, *SLC2A3*, *PIK3R1*, *PIK3R2*, *PGF*, *RRAS2*, and *EGLN3*. Meanwhile, in the tongue, fewer genes are expressed that relate to the HIF-1α pathway (e.g., *MMP10*, *SLC2A1*, *PIK3R1*, and *PIK3R5*) [186]. Fewer keratinocytes lead to less scar tissue formation in oral wounds than in skin wounds [197]. This is due to increased levels of *SOX2* and *PITX1* expression by primary human oral cells, which are unique to the oral cavity, and they accelerate wound closure [198]. In contrast, expression of α-SMA (myofibroblasts) is higher in oral sites of injury than in skin sites of injury during the late stages of wound healing. Expression of α-SMA is followed by elevated levels of TGF-β and pSmad3 [199]. The essential role of TGF-β/Smad3 signaling in wound healing [200] is consistent with the motility and infiltration of monocyte macrophages to wound areas. The former has been demonstrated in both *Smad3*-deficient mice and with use of *Smad3*-targeted siRNA, where acceleration in palatal wound repair was achieved [201,202]. Furthermore, the observed acceleration was accompanied by decreased expression of TGF-β, α-SMA, MCP-1, and MIP-1 α [201,202]. In a study of macrophage recruitment to oral wound sites vs. skin wound sites, it was confirmed that macrophages arrive earlier to oral wounds than to skin wounds, then were reduced in number to the level of unwounded tissue after 60 days [199]. Meanwhile, the number of macrophages at skin wounds remained high after 60 days [199]. Based on these results, it appears that a low number of macrophages (CD68, CD40, CD206, CD163) in oral wounds [195] may be beneficial for achieving scarless formation since they are known to abundantly produce more pro-fibrotic TGF-β1 in oral wounds than in skin wounds [199,203].

It is also possible that rapid oral wound healing is facilitated by the presence of saliva/mucous in the oral cavity, which can provide many necessary cytokines, growth factors, and protease inhibitors [193,204,205]. However, it is recognized that saliva alone is not responsible for the rapid healing of oral mucosal wounds. Rather, tissue characteristics, location, size, type of injury, salivary flow, microflora, and temperature are factors that also need to be considered [193]. Thus, macrophages provide critical, multi-faceted functions in wound repair by acting as pro- or anti-inflammatory agents. Given the importance of their role, the functions of macrophages in the oral mucosa remain to be further explored.

Several wound healing treatments related to oxygen therapy have been developed, which mediate anti-inflammatory effects. These include supplemental oxygen therapy and molecular hydrogen as an antioxidant, which are applied in preventive and therapeutic applications [186,206]. In both skin and mucosal wounds, it has been observed that hyperbaric oxygen therapy does not preserve HIF-1α and VEGF expression [186]. Consequently, no acceleration in wound closure is predicted. In palatal wound closures, hydrogen-rich water has been effective in accelerating healing via upregulation of the nuclear factor E2-related factor (Nrf)-2/antioxidant defense pathway. The latter is characterized by increases in heme oxygenase-1 and NAD(P)H quinone dehydrogenase-1 and reduced levels of iNOS [206]. Increased levels of healing-associated genes (e.g., *FGF7*, *VEGF*, *TGF-1β*, *α-SMA*) have also been observed [206]. 

An important mediator for skin and mucosal wound healing is IL-33, a potent Th2-type immune response [207,208,209]. Administration of IL-33 effectively accelerates wound healing by shrinking the wound area and increasing re-epithelialization and accumulation of collagen and fibronectin [207]. With an abundance of collagen and fibronectin, greater scar thickness is possible. IL-33 is also associated with an increased number of M2 macrophages (CD206), arginase-1 expression, and alternatively activated macrophages [207].

### 4.3. Cross Signaling among HIF-1α/S1P/S1PRs in Macrophages

Monocytes/macrophages are tightly dependent on HIF-1α expression in wound healing [210]. HIF-1α recruits monocytes/macrophages by promoting CCL2/MCP-1 secretion and also has a strong correlation with CD68 expression [211]. A hypoxic environment modulates macrophage functions both directly (oxidase, oxygenase, and hydroxylase) and indirectly (ATP/AMP, ROS, Krebs cycle, and Fe^2+^ levels) [212,213,214]. Meanwhile, S1P dependently affects chemotaxis of M1/M2 polarization without influencing phagocytic activity [53]. A hypoxic wound environment causes SphK1 to be rapidly activated via ROS, and this precedes accumulation of HIF-1α [90]. On the other hand, an increase in intracellular S1P occurs due to the conversion of ceramide to sphingosine, which is activated by HIF-1α via ERK, Akt, and the GSK3β pathway [215]. Thus, SIP activates and enhances the lifespan of macrophages [90,98]. In addition, S1P and adenosine [216] act to regulate HIF-1α production by controlling gene expression of *VEGF* and *TGF-α* through S1P_3_, G_i_, and their downstream effectors (PKC-β1, MEK, PI3K, and mTOR) under normoxic conditions [56].

In macrophages, long-term hypoxic stress (chronic inflammation) upregulates Toll-like receptor (TLR)-4 mediated HIF-1α through the PI3K/Akt pathway, yet not via p38 [217,218]. Moreover, the PI3K/Akt pathway stabilizes HIF-1α via inhibition of GSK3β in the early stages of hypoxia [104]. When Semba et al. characterized the migration and activation of macrophage during the early stages of systemic inflammation, they found that HIF-1α-induced pyruvate dehydrogenase kinase (PDK)-1 promotes glycolysis, while cytochrome *c* oxidase remains unaffected [219]. In addition, it was observed that cross talk between HIF-1α and S1P/S1P_1_ signaling controls the migration of M1/M2 macrophages to a wound site via the downstream effectors ERK, Akt, and p38 [57]. Correspondingly, reduced macrophage migratory activity is associated with HIF-1α deficiency [57,219]. ATP is rapidly consumed in the cytosol due to the remodeling of cytoskeletal actin filaments that occurs with migration [220,221]. Thus, it is possible that cross talk exists between glycolytic reprogramming and actin filament remodeling during activation of macrophage migration under hypoxic conditions.

Interestingly, administration of dimethyloxalylglycine (DMOG), an inhibitor of PHD and α-ketoglutarate, helps accelerate wound closure and upregulates macrophage migration in a partial HIF-1α KO mouse model [57]. DMOG activates AMPK [222], HIF-1α, VEGF [223], and NF-κB [138,224], thereby abrogating pro-inflammatory cytokine expression. DMOG also activates PGC-1α to increase mitochondrial activity and glycolytic flux [225,226]. Endothelial-specific S1P_1_ KO mice exhibit a defect in vascular stabilization and systemic failure due to upregulation of *VEGF* by HIF-1α [227]. Lim et al. investigated a wound healing treatment involving the PHD inhibitor ciclopiroxolamine (CPX) and S1P [228]. In vitro, CPX and S1P extensively affected endothelia and the fibroblast formatting vascular network by expressing MCP-1 and VEGF [228]. Thus, stabilizing and inducing HIF-1α gene expression by inhibiting mitochondrial activity [224] can potentially enhance cellular sensitivity to S1P via Rho family GTPase Rac activation [228]. Thus, the HIF-1α/S1P_1_ signaling axis [57] [227] may represent a key regulator of inflammation not only in myeloid cells [185] but also in fibroblasts and endothelial cells [228].

## 5. Conclusions

In summary, the HIF-1α/S1P/S1PR signaling axis appears to play a critical role in the activation and polarization of macrophages, and this contributes to the inflammatory conditions at a wound site. However, additional studies are needed to better understand the exact role of S1P-induced functional consequences for macrophage biology in different wound healing entities, especially with respect to targeting the HIF-1α/S1P/S1PR signaling axis as a strategy for wound healing therapy.

## Figures and Tables

**Table 1:** S1P/S1PRs signaling and monocyte/macrophage function under inflammatory and pathological conditions.

Inflammatory Conditions	Signaling Pathways	S1P Receptors	Roles
Palatal wound healing [57]	MCP-1, HIF-1α, MIP-1α, iNOS, TNF-α, Akt, p38	S1P_1_	Infiltration of M1/M2 macrophages
CNS autoimmunity and neuroinflammation [41]	STAT-3, JAK, IL-6	S1P_1_	Pronounced activation of monocytes
Inflammation and atherosclerosis [21,24,25,31,32,33]	TNF-α, MCP-1, IL-6, Akt, PI3K, PKC, ERK1/2, p38, Rho kinases	S1P_1_	Activation and migration of macrophages
	Gα_12/13_, NF-κB, RhoA	S1P_2_	Inhibits macrophage migration (negative effect) and inhibits M1 activation
	MCP-1, TNF-α, Sphk1	S1P_3_	Migration of monocytes/macrophages
Tubulointerstitial inflammation [36]	MCP-1, SphK1, TNF-α, arginase-1, IL-6, IL-10	S1P_1_ S1P_3_	Macrophage infiltration
Acute allergic [37]	MCP-1/CCL2, MIP-1α, RANTES/CCL5	S1P_2_	Macrophage infiltration
Inflammation and liver injury [29,30,179]	Gα_i/o_, PI3K, JNK, Rac1	S1P_2_ S1P_3_	Migration of BMMs, M1 polarization
	NLRP3, IL-1β, IL-18, p38, ERK, JNK, Gα_12/13_	S1P_2_	Blockade BMMs activation and M1 polarization
Rheumatoid arthritis [23,175]	NF-κB, Fas, Akt, p38, ERK1/2, Rac, Rho	S1P_1_	Motility and number of BMMs induced osteoclast
Cerebral ischemia [49]	NF-κB p65, ERK1/2, p38, Akt	S1P_3_	M1 polarization
Bacterial sepsis [50,173,177]	Caspase-11	S1P_2_	Macrophage pyroptosis
	MCP-1, Sphk1, TNF-α, arginase-1, IL-6, IL-10	S1P_1_ S1P_3_	Macrophage infiltration
Psoriasis [54]	IL-6, CCL2, CXCL1	S1P_4_	M2 macrophage infiltration
Bone marrow sinusoidal inflammation [55]	CCR2	S1P_5_	Monocyte migration
Pulmonary disease [27,28]	CCR2	S1P_5_	Monocyte migration
Encephalomyelitis [25]	CCR2	S1P_5_	Inflammatory monocytes supply the concentration of lymph node S1P

Several inflammatory conditions related to monocyte/macrophage disturbances (wound healing, allergies, liver injury, and arterial/pulmonary diseases) are a hallmark feature of hypoxia and are detected by S1P/S1PR signaling. These disturbances are related to various cellular signaling pathways that mediate cell survival, migration, and apoptosis. S1P_1–3_ contribute to the activation, motility, and infiltration of monocytes/macrophages. Meanwhile, S1P_4–5_ only contribute to the migration and infiltration of monocytes/macrophages. However, supporting research remains to be further analyzed. Thus, activation of S1P/S1PRs in monocytes/macrophages through various pathways can trigger functional responses as indicated. Targeting of the genes indicated may therefore be of interest in disease settings.
